# Mothers' and Fathers' Experiences of Family Relations and Parenting During the First Year of Parenthood

**DOI:** 10.1002/pchj.823

**Published:** 2025-01-23

**Authors:** Tatiana G. Bokhan, Svetlana B. Leshchinskaia, Olga V. Terekhina, Marina V. Shabalovskaya, Anna V. Silaeva, Sergey B. Malykh, Yulia Kovas

**Affiliations:** ^1^ Department of Psychology National Research Tomsk State University Tomsk Russia; ^2^ Psychological Institute Russian Academy of Education Moscow Russia; ^3^ The Centre for Research on Intelligence and Cognitive Well‐Being, Institute for Cognitive Neuroscience Higher School of Economics Moscow Russia

**Keywords:** family relations, infancy, marital relations, measurement invariance, parenting

## Abstract

The first year of parenthood is considered to be a challenging period, associated with the transformation of family relations. The links between family relations and parenting are widely studied. However, in most research only a limited number of indicators is investigated, and there is a lack of data on the agreement between mothers' and fathers' evaluations of family relations. The aims of the present study were to explore (1) the structure and measurement invariance of marital relations and parenting constructs for mothers and fathers; (2) the associations among the measures of marital relations and parenting in mothers and fathers; (3) the agreement between mothers and fathers in their perception of marital relations, as well as cross‐parent cross‐measure associations of marital relations and self‐rated parenting; (4) average differences between the parents in their perception of marital relations and parenting. The data from 352 Russian‐speaking married couples participating in the Wave 3 of the Prospective Longitudinal Interdisciplinary Study (PLIS) were collected when the children were 9 months old. Seven measures of family relations (marital relations, grandparents' support) and nine measures of parenting were obtained. The statistical analyses included the exploratory factor analysis, assessment of measurement invariance, comparative and correlational analysis. The result showed that measures were organised into coherent factor‐based groupings: (1) marital relations, (2) support from grandparents, (3) childcare and affection, and (4) harsh parental discipline. Six of 12 measures showed partial scalar invariance between mothers and fathers. Moderate within‐measure correlations were observed between mothers' and fathers' assessments of family relations; and weak correlations—for parenting. Mother‐father cross‐measure correlations were moderate for family relations, but negligible for parenting. Small to moderate average differences between mothers and fathers were found for all measures. The results highlight the need to consider similarities and differences between mothers' and fathers' experiences in future research and practice.

## 1 Introduction

Family relations are the core of the family life. The importance of research investigating various aspects of relationships in a family, including marital and parent‐child relations, stems from their well‐established links with parental and child wellbeing, for example, parental mental health (Kouros and Cummings 2011; Peterson‐Post et al. 2014; Kestler‐Peleg et al. 2020; Pieh et al. 2021; Çankaya and Alan Dikmen 2022), children's mental health (Borelli, Margolin, and Rasmussen 2015; Rajyaguru et al. 2019; Marchand‐Reilly and Yaure 2019; Li et al. 2020), behaviour of children (Goldberg and Carlson 2014; Bornstein, Putnick, and Suwalsky 2018; Park and Dotterer 2018; Parkes, Green, and Mitchell 2019) and children's cognitive development (Gibbs and Forste 2014; Sethna et al. 2017; Binda, Figueroa‐Leigh, and Olhaberry 2019; Berthelon et al. 2020). Increasingly, research has focused on the importance of fathers' role in child development (Lamb 2010; Volling et al. 2019) and the need to explore potential differences between maternal and paternal attitudes to parenting (Palkovitz, Trask, and Adamsons 2014; Crapo et al. 2021). Other family members may also contribute to the system of family relations effects. For example, there is evidence that relations with grandparents can moderate negative effects of parenting (Akhtar, Malik, and Begeer 2017; Sorek 2020).

### 1.1 Associations Between Family Relations and Parenting of Infants

The first year after the birth of the child is associated with significant changes in the family structure (LeMasters 1957) and transformation of family relations (Kluwer 2010; Delicate, Ayers, and McMullen 2018). During this period family relations gain additional significance, as they may influence parents' attitudes and interactions with the infant. For example, one meta‐analysis reported associations between inter‐parental conflict and harsh parenting practices, with moderate effect size (Krishnakumar and Buehler 2000). In this meta‐analysis, the ‘spillover model’ of this relationship was suggested, indicating that emotions caused by marital difficulties tend to be expressed in parental interactions with the child. Another study observed that fathers' prenatal marital satisfaction significantly predicted their involvement in childcare when the child was 6 and 12 months old, with small effect (Lee and Doherty 2007). One study found that paternal marital satisfaction moderately predicted the quality of father–son interaction, whereas for daughters this interaction was not significant (Bernier, Jarry‐Boileau, and Lacharité 2014).

In turn, parental experiences of interaction with the infant may have an impact on family relations. One study showed that for fathers, self‐efficacy in parenting and perceived level of parenting alliance significantly moderated the relationship between stress related to fathering and marital satisfaction, with moderate effect size (Kwan, Kwok, and Ling 2015). Another cross‐sectional study reported that better co‐parenting alliance, higher satisfaction with father involvement and lower level of parenting stress predicted higher maternal marital satisfaction, with medium effect size (Kwok et al. 2015).

Few studies used multiple measures of family relations and parenting to explore associations among these processes in both mothers and fathers. One study found significant inter‐parental associations between positive relationship behaviours and marital satisfaction (Rauch‐Anderegg et al. 2020). The relationship between attachment to the partner and marital satisfaction was found to be mediated by quality of communication between the partners, with stronger effect on wives than on husbands (Hou, Jiang, and Wang 2019). Another study reported complex links between marital conflict and parenting (Gao et al. 2019). Fathers' stonewalling behaviours in conflict (withdrawal, refusal to communicate) significantly predicted mothers' unsupportive reactions to children's negative emotions. Mothers' destructive conflict behaviours (stonewalling and aggression) predicted less punitive maternal reactions to children's negative emotions, though the effect sizes were small. Another study found significant modest association between the level of mother‐reported marital conflict and differences in perceived authoritativeness between mothers and fathers (Tavassolie et al. 2016). In other words, the more parents differed in their authoritativeness towards the child, the greater conflict mothers experienced in marital relationships. One recent study showed small significant negative correlation between fathers' relationship satisfaction during the pregnancy and infancy and fathers' reflective parenting skills (characterised by the ability to understand their own and children's feelings and thoughts) when the child was 3 months. These relationships were not significant in mothers (Salo et al. 2022).

### 1.2 Agreement Between Mothers and Fathers' Experiences of Family Relations and Parenting

A number of studies examined the strength of agreement between mothers' and fathers' experiences of family relations and parenting. For example, one study found only moderate correlations for mothers' and fathers' marital satisfaction when the child was 4 and 12 months old (Elek, Hudson, and Bouffard 2003). Another study found moderate intra‐couple correlations for parental efficacy and over‐reactive parenting at child's 9, 18 and 27 months (Lipscomb et al. 2011).

More research is available beyond the first year of life, reporting moderate to strong correlations of different aspects of marital relations between parents, including family functioning (Pisula and Porębowicz‐Dörsmann 2017), constructive and destructive marital conflict (McCoy et al. 2013). Significant moderate positive intra‐couple correlations were observed for different measures of parenting, including: for authoritative, authoritarian and permissive parenting styles in parents of toddlers (Rinaldi and Howe 2012; Tavassolie et al. 2016); supportive parenting of 6–9 years old children (Slagt et al. 2012); parental support and control for elementary school children (Kuppens and Ceulemans 2019); harsh parenting of preschool (Lucassen et al. 2015) and elementary school (Kuppens and Ceulemans 2019) children.

### 1.3 Average Differences Between Mothers and Fathers' Experiences

Although the childbirth is a critical period for the whole family as a system, the challenges parents experience may differ, based on the involvement in childcare, general attitudes towards parenting, the need to take parental leave, societal expectations, and so forth (LeMasters 1957). For example, women may on average experience greater role conflict and freedom restrictions caused by parenting than men, as they are traditionally facing greater role changes on becoming mothers (Twenge, Campbell, and Foster 2003). Therefore, the perceptions of marital relations may differ between mothers and fathers. Parenting behaviours and perceptions can also differ, as mothers and fathers on average differ in the types of activities performed with children, and the amount of time spent with their children, especially in traditional societies (Craig 2006; Chiorri et al. 2015). Research has also suggested that women on average experience higher tiredness and stress while performing childcare activities, which may add to the differences in perception of overall family situation (Connelly and Kimmel 2014).

Empirical research found a number of substantial differences in mothers' and fathers' perceived family relations and parenting. For example, several studies found that mothers of infants reported significantly higher relationship satisfaction than fathers (Twenge, Campbell, and Foster 2003; Nicolaus et al. 2021). In another study, no significant differences were found between mothers and fathers in perceived emotional quality of marital relations, but mothers reported greater emotional quality of parent–child relationship (Kouros et al. 2014). No differences between parents of infants in the perception of their impact on child's future development and of child's personal qualities were observed (Boivin et al. 2000). In contrast, mothers of infants reported significantly higher self‐efficacy in parenting than fathers, more affection and warmth towards the child, greater tendency for overprotection and lower harsh parenting (Boivin et al. 2000). Mothers also demonstrated greater sensitivity in parenting compared to fathers (Barnett et al. 2008).

### 1.4 Measurement Invariance for Mothers and Fathers

Only limited research examined measurement invariance between mothers and fathers, although the research suggests there are differences in marital satisfaction (Twenge, Campbell, and Foster 2003) and parental roles and activities (Hay et al. 1999; Chiorri et al. 2015) that may be a source of potential invariance. Most of these studies found invariance or partial invariance for many aspects of parenting, including autonomy support (Hughes, Lindberg, and Devine 2018); parental beliefs on facilitation of children's independence (Crapo et al. 2021); acceptance, psychological intrusiveness, parental harshness (Adamsons and Buehler 2007); parental warmth (Crapo et al. 2021).

Overall, the existing theoretical and empirical work suggests that mother, father and infant form a complex family system of co‐influencing elements, and that it is important to better understand both mothers' and fathers' perceptions of family relations and parenting. To date, the relevant research has been largely limited to Western samples and very few studies explored multiple measures and their interrelations in both parents. It is important to examine different aspects of marital relations, such as overall perception of the quality of family relations, perceived marital satisfaction, characteristics of marital interaction and perceived support from a spouse. Previous research suggests that childbirth may be associated with a decrease in marital quality and satisfaction, and with greater conflict (Twenge, Campbell, and Foster 2003; Lawrence et al. 2008; Castellano et al. 2014). Moreover, perceived support from a spouse and marital satisfaction are associated with mental health and wellbeing after childbirth (Japel, Tremblay, and McDuff 2000; Boyce and Hickey 2005; Iles, Slade, and Spiby 2011; Lokubal et al. 2021) and with harsh parenting behaviours (Boivin et al. 2000). Previous literature also identified support from grandparents as an important influence in child development (Buchanan and Rotkirch 2018; Sadruddin et al. 2019) and showed high involvement of grandparents in childrearing in most Russian families (Sivak 2018). In addition, it is important to explore multiple characteristics of parenting of the infant, such as perceived self‐efficacy, perceived impact on child's development, coercive behaviours, warmth, overprotection, overall perception of child's qualities, parenting practices. Together they tap into the most important aspects of family relations during the first months of parenting (Boivin et al. 2000; Castellano et al. 2014)—indexing the overall quality of parenting which is linked to child's further development (Boivin et al. 2000).

### 1.5 The Present Study

The present study aims to provide new knowledge by employing a comprehensive assessment strategy, using multiple measures of family relations and parenting in a sample of mothers and fathers. Specifically, we explored:

1. Structure and measurement invariance of the marital relations and parenting constructs for mothers and fathers.
2. Associations among the measures of marital relations and parenting in mothers and fathers.
3. Agreement between mothers and fathers in their perception of marital relations, as well as cross‐parent cross‐measure associations of marital relations and self‐rated parenting.
4. Average differences between the parents in their perception of marital relations and self‐rated parenting, including when controlling for parental agreement.

In order to address these questions, we used data collected at 9 months after the childbirth in families with natural conception and conception via assisted reproduction technology treatment (ART), as part of Prospective Longitudinal Interdisciplinary Study (PLIS) in Russia.

## 2 Method

### 2.1 Participants

Participants were part of the ongoing study PLIS in Russia that began in 2015. PLIS is based on the design and methods used in QLSCD (The Québec Longitudinal Study of Child Development), C‐IVF (Cardiff IVF Study) and TEDS (Twins Early Development Study). The study investigates two groups of families (mothers, fathers and their children): naturally conceiving families and those who conceived through ART. The participants are recruited via prenatal medical centres during the 1st trimester of pregnancy and assessed at eight timepoints: 2nd and 3rd trimesters of pregnancy, child's age 9, 18, 29 months, 4, 5 and 6 years (see Voronina et al. 2016 further details). The research was approved by the Ethics Committee for Interdisciplinary Studies of Tomsk State University, and written informed consent was obtained from the participants.

As families are recruited on a rolling basis, the data are analysed from each wave when the required participant number is reached. Further longitudinal analyses are planned once the data collection is complete from all waves. Here, we present the data from the 3rd research wave, which was 9 months after the childbirth. These data were collected in 2016–2020 with the research booklets sent to participants by post. About 29.3% of the families participated in the study in 2016, 47.3%—in 2017, 14.9%—in 2018; 7%—in 2019; 1.4%—in 2020. Therefore, most data were collected before the COVID‐19 pandemic.

In total, the data from 352 Russian‐speaking married couples were collected: 238 families with natural conception and 114 couples with conception via ART; mean age of mothers *M* = 31.64, *SD* = 4.73; fathers: *M* = 33.25, SD = 5.64. As mothers or fathers from some families did not participate in the study, the paired data were available for 324 couples. The majority of mothers (82%) and fathers (62.5%) completed higher education, 15% of mothers and 26% of fathers received professional training, 3% of mothers and 11.5% of fathers had secondary school education. 92% of mothers were on maternity leave in the period of assessment, and 98% of fathers were employed (full time—79%, part‐time—2%, shift work—5%, self‐employment—12%).

### 2.2 Procedure and Measures

Each parent completed a booklet when their child was 9 months old. In the present study, we used data collected using seven established and validated questionnaires which had been used in previous longitudinal research (e.g., QLSCD and C‐IVF). The measures were adapted for administration in the Russian speaking sample according to the ITC guidelines for test translation (International Test Commission 2017).

**FIGURE 1:**
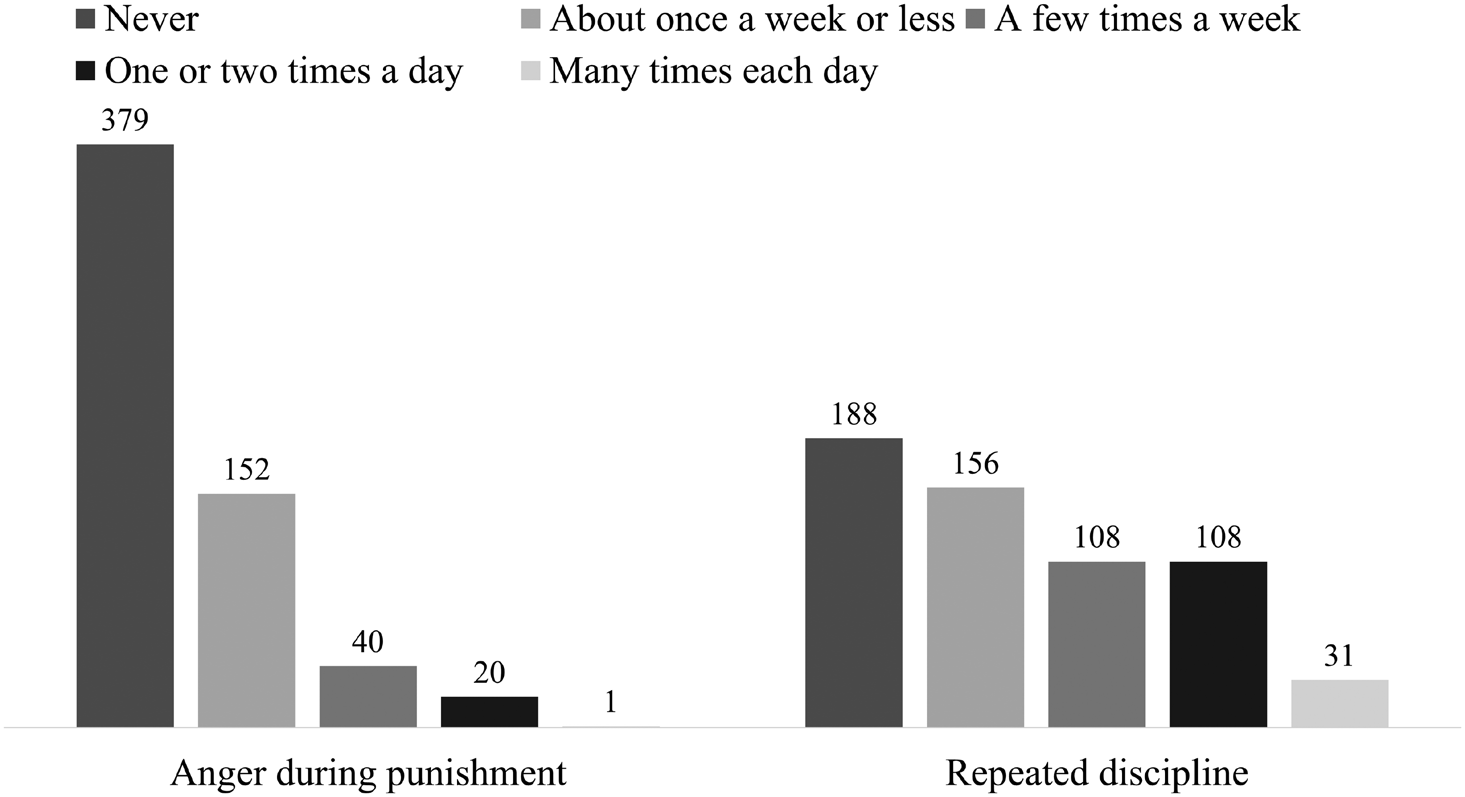
Distribution of the *negative practices* scale items on the whole sample.

Each of the seven questionnaires contained several scales (see Table 1). The internal consistency for all scales was estimated by Cronbach alpha. As can be seen from Table 1, the alphas ranged from 0.63 to 0.90, which is acceptable to good according to published recommendations (Janssens et al. 2008; Hajjar 2018). One exception was the negative practices scale of the parenting practices construct, which showed very low internal consistency (0.41). We checked the distributions of the questions included in this scale (see Figure 1): Item 1 ‘How often do you get angry when you punish your baby?’ and Item 2 ‘How often do you have to discipline him/her repeatedly for the same thing?’

**TABLE 1:** Measures of family relations and parenting.

Title	Construct and subscales (Cronbach's alpha)	Items no. (max score)	Scoring
Family relations			
General Functioning scale of the McMaster Family Assessment Device (MFAD, Epstein, Baldwin, and Bishop 1983)	1. Current family relationships^a^: quality of family relations (0.86)	12 (48)	A 4‐point Likert scale: *completely disagree*—1, *totally agree*—4 Overall family functioning was calculated as the sum of all answers. According to Akister and Stevenson‐Hinde (1991), scores below two indicate healthy family functioning. For the purpose of our studies, the scale was reversed, so that higher scores indicated better functioning
Feeling of happiness in the relationship during the last 9 months scale spart of The Locke‐Wallace Marital Adjustment Scale (LWMAS; Locke and Wallace 1959)	2. Perceived level of happiness in the relationship Participants are told that the score of 4 (‘happy’) corresponds to the degree of happiness most people get from partner relationships^b^	1 (7)	A 7‐point scale: *very unhappy*—1, *perfectly happy*—7
IOWA Family Interaction Rating Scales (Melby and Conger 2001)	Family interaction: emotional warmth and hostility towards the spouse Scales: 3. Warmth towards the partner (0.90) 4. Hostility towards the partner (0.84)	10 (70)	A 7‐point scale (*never*—1, *always*—7). For each of the partners warmth and hostility were calculated as a sum of the corresponding items Overall score of relationship was calculated as a sum of warmth and reversed hostility scales; with higher scores indicating better relations
Support from the Partner (see Thibault, Jetté, and Desrosiers 2001)	5. Instrumental and psychological support from the partner (0.90)	5 (50)	A 10‐point scale (0—never helps, 10—always helps). The overall score was calculated as a sum of all items
Support from the infant's grandparents (see Thibault et al. 2003)	Support from the infant's grandparents (on the mother's and father's side) Scales: 6. Support from own parents (0.76) 7. Support from spouse's parents (0.74)	10 (58)	Frequency of financial support was assessed on a 5‐point scale (1—never, 5—often) Next four questions (support with household chores; childcare; psychological support; advice on child development) were assessed on a 6‐point scale (1—never; 6—every day) All scales were completed for respondent's parents' and spouse's parents' The overall grandparents' support was calculated as the sum of all answers
Parenting			
Parental Perceptions and Behaviors Regarding the Infant Scale (PPBS; Boivin et al. 2000)	Overall relationship with the infant: feelings, thoughts and actions towards the child^a^ Scales: 8. Feeling of self‐efficacy (0.77) 9. Perception of impact^a^ (0.77) 10. Tendency to coercion (0.79) 11. Affection (0.79) 12. Overprotection (0.63) 13. Perception of the child's qualities (0.75)	32 (320)	A 10‐point Likert scale (0—*absolutely wrong*, 10—*absolutely true*) Each scale and the total score were calculated as the sum of the items. The higher score indicated higher intensity of the corresponding dimension Number of items: (a) 6; (b) 5; (c): 7; (d) 5; (e) 5; (f) 4
The short 6‐item version of the Positive/Ineffective Parenting Practices scales (see Thibault, Jetté, and Desrosiers 2001)	Frequency of the positive and ineffective practices towards the child Scales: 14. Positive practices (0.90) Negative practices (0.41) assessed as separate items: 15. Anger during punishment 16. Repeated discipline	6 (30)	A 7‐point Likert scale: *never* (1 point), *about once a week and less* (2 points), *several times a week* (3 points), *once or twice a day* (4 points), *many times a day* (5 points) Number of items: (a) 4; (b) 2

*Note:* The Cronbach's alpha is not presented.

^a^ The reverse items were transformed.

^b^ The scale consists of one item.

The distribution of the two items was skewed to the left, indicating that these situations were rare in this sample. In addition, we performed the Spearman rank correlation analysis to explore how these two items relate to a similar construct (tendency to coercion), which is included in the Parental Perceptions and Behaviours Regarding the Infant Scale. Correlations were: 0.28 (*p* < 0.01) between Item 1 (anger during punishment) and Item 2 (repeated discipline); and 0.57 (*p* < 0.01) between Item 1 and tendency to coercion; 0.17 (*p* < 0.01) between Item 2 and tendency to coercion. Based on these analyses, it was decided to include Items 1 and 2 as individual items.

In total, 16 scales were included in further analyses (see Table 1): five scales assessing all aspects of couple relations (current family relationships, happiness in the relationship, warmth towards the partner, hostility towards the partner, support from a partner); two scales measuring grandparents' support (support from own parents and support from partner's parents); nine scales assessing parenting of an infant (self‐efficacy, perceived impact on child's future development, coercive parental behaviours, feelings of affection towards the child, overprotection, perception of child's unique qualities, positive parenting practices, negative parenting practices 1 [anger during punishment], negative parenting practices 2 [the need to discipline the child repeatedly for the same acts]).

### 2.3 Data Analysis

Here we present the analysis conducted on the combined pregnancy types group (natural conception and ART) to enhance the statistical power for exploring agreement and differences between mothers and fathers. Previous comparisons between natural conception and ART groups found no differences or only small differences on similar variables collected during pregnancy (Feklicheva et al. 2017; Bokhan et al. 2018, 2021). We plan to explore potential group differences on longitudinal data in our future work, as there are inconsistent findings in the literature (e.g., Hahn 2001; McGrath et al. 2010).

The normality was assessed based on skewness and kurtosis values (acceptable range +/−2); and internal consistency was assessed using Cronbach's alpha test.

First, we explored structure and measurement invariance of the marital relations and parenting constructs for mothers and fathers. We conducted factor analyses in order to obtain the information on the latent constructs that reflect the inner structure of various aspects of relations and parenting. We conducted the exploratory factor analysis (EFA) using principal axis factoring with varimax rotation to obtain the model for further confirmatory factor analysis (CFA). All 16 subscales were included in the EFA, and only the items with loading higher than 0.40 were included in the final model. The structure was first calculated on a whole sample, and then on mothers and fathers separately. The factor solution was chosen based on a scree plot and total variance explained (higher than 50%; Samuels 2017). The model that was valid for both groups was chosen for further testing.

We then assessed measurement invariance of these measures for mothers and fathers. These analyses utilise the available data from two informants and provide the necessary information for interpretation of the main results, as well as for understanding of similarities and differences in the meaning of latent constructs for mothers and fathers (Widaman and Reise 1997; Putnick and Bornstein 2016; Crapo et al. 2021). We performed CFA to assess measurement invariance at three steps: configural, metric and scalar (Putnick and Bornstein 2016). The configural invariance was assessed based on CFI values (> 0.9), RMSEA (< 0.08), ΔCFI (< 0.01) and Δ*χ* ^2^ (non‐significant) (Cheung and Rensvold 2002). Based on these analyses, we created composite measures in order to reduce the number of statistical tests (Wootton et al. 1997; Spokas and Heimberg 2008). For further analyses, we used the composite measures consisting of those subscales that showed good scalar invariance for mothers and fathers. In addition, the scales that were non‐invariant were treated as separate measures.

Second, we explored associations among marital relations and parenting, separately for mothers and fathers, using correlation analysis.

Third, we explored the extent to which mothers and fathers agree in their evaluations of family relations. Also, we assessed the cross‐measure associations between mothers' and fathers' perceptions of family relations and parenting. Pearson's correlations were used for these analyses.

Fourth, we compared means for fathers and mothers using the paired *t*‐test or the Wilcoxon signed rank test, depending on the normality of distribution. For measures of marital relations, we additionally performed ANCOVA to check whether the differences in the variables obtained after invariance analysis can be explained by the differences in the perception of marital relations. To test this, we added the *differences in the perception of current family relationships* score (computed as mothers' minus fathers' *current family relations* score) as a covariate. This measure was chosen because it allows to detect the discrepancy on the perception of the same family situation by spouses. The dependent variables were *marital well*‐*being* (combined happiness in the relations and reversed hostility), *current family relationships* and *warmth towards the partner*.

The statistical analyses were performed with the IBM SPSS Statistics Version 26.0 and IBM SPSS Amos 23.0.

## 3 Results

Descriptive statistics of all measures in groups of mothers and fathers are presented in Table A1.

### 3.1 Structure and Measurement Invariance in Marital Relations and Parenting in Mothers and Fathers

The results of the EFA on combined sample of mothers and fathers are presented in Table 2. Scales *perception of impact*, *perception of the child's qualities* and item *repeated discipline* did not load significantly on any of the factors. Therefore, the structure of family relations and parenting was represented by 4 factors with 13 scales with loadings > 0.40. The four factors together explained 65.01% of the variance. Two factors, related to family relationships, reflected the scales of the original questionnaires: (1) ‘marital relations’ (positive family functioning, feeling of happiness in the relations, warmth expressed towards the partner and perceived support from the partner); (2) ‘Support from grandparents’ (infant's maternal and paternal grandparents). Two factors characterised aspects of parenting: (3) ‘childcare and affection’ (feeling of self‐efficacy in parenting, affection towards the child, overprotection and positive parental practices); and (4) ‘Harsh parental discipline’ (punishment and negative emotions in parenting).

**TABLE 2:** EFA of family relations and parenting measures (whole sample).

Dimensions	% of variance explained	Scales	Factors			
			1	2	3	4
1. Marital relations	27.86	1. Current family relationships	0.68			
		2. Happiness in the relationship	0.71			
		3. Warmth towards the partner	0.75			
		4. Hostility towards the partner	−0.72			
		5. Support from a spouse	0.63			
2. Support from grandparents	10.67	6. Support from own parents		0.45		
		7. Support from spouse's parents		0.73		
3. Childcare and affection	17.36	8. Feeling of self‐efficacy			0.72	
		11. Affection			0.75	
		12. Parental overprotection			0.45	
		14. Positive practices			0.66	
4. Harsh parental discipline	9.12	10. Tendency to coercion				0.76
		15. Anger during punishment				0.70

*Note:* Extraction method: principal axis factoring. Rotation method: varimax with Kaiser normalisation.

We then performed the factor analysis on data from mothers and fathers separately (Table 3). The structure of the factors ‘marital relations’, ‘support from grandparents’ and ‘harsh parental discipline’ was equal for the two groups. However, the structure of the factor ‘childcare and affection’ differed between mothers and fathers. The scale *overprotection* loaded on ‘childcare and affection’ factor for fathers, and did not load significantly on any of the factors for mothers.

**TABLE 3:** EFA in mothers and fathers groups.

Dimensions	Mothers	Fathers
The total percent of the variance explained by the factors in the exploratory factor analysis	64.21	63.16
1. Marital relations	1. Current family relationships (0.80)	1. Current family relationships (0.70)
	2. Happiness in the relationship (0.76)	2. Happiness in the relationship (0.58)
	3. Warmth towards the partner (0.73)	3. Warmth towards the partner (0.73)
	4. Hostility towards the partner (−0.72)	4. Hostility towards the partner (−0.69)
	5. Support from a spouse (0.64)	5. Support from a spouse (0.58)
2. Support from Grandparents	6. Support from own parents (0.58)	6. Support from own parents (0.87)
	7. Support from spouse's parents (0.50)	7. Support from spouse's parents (0.53)
3. Childcare and affection	8. Feeling of self‐efficacy (0.67)	8. Feeling of self‐efficacy (0.61)
	11. Affection (0.76)	11. Affection (0.84)
	**—**	12. Parental overprotection (0.41)
	14. Positive practices (0.46)	14. Positive practices (0.52)
4. Harsh parental discipline	10. Tendency to coercion (0.75)	10. Tendency to coercion (0.68)
	15. Anger during punishment (0.68)	15. Anger during punishment (0.71)

*Note:* Extraction method: principal axis factoring. Rotation method: varimax with Kaiser normalisation. Factor loadings are presented in brackets.

**FIGURE 2:**
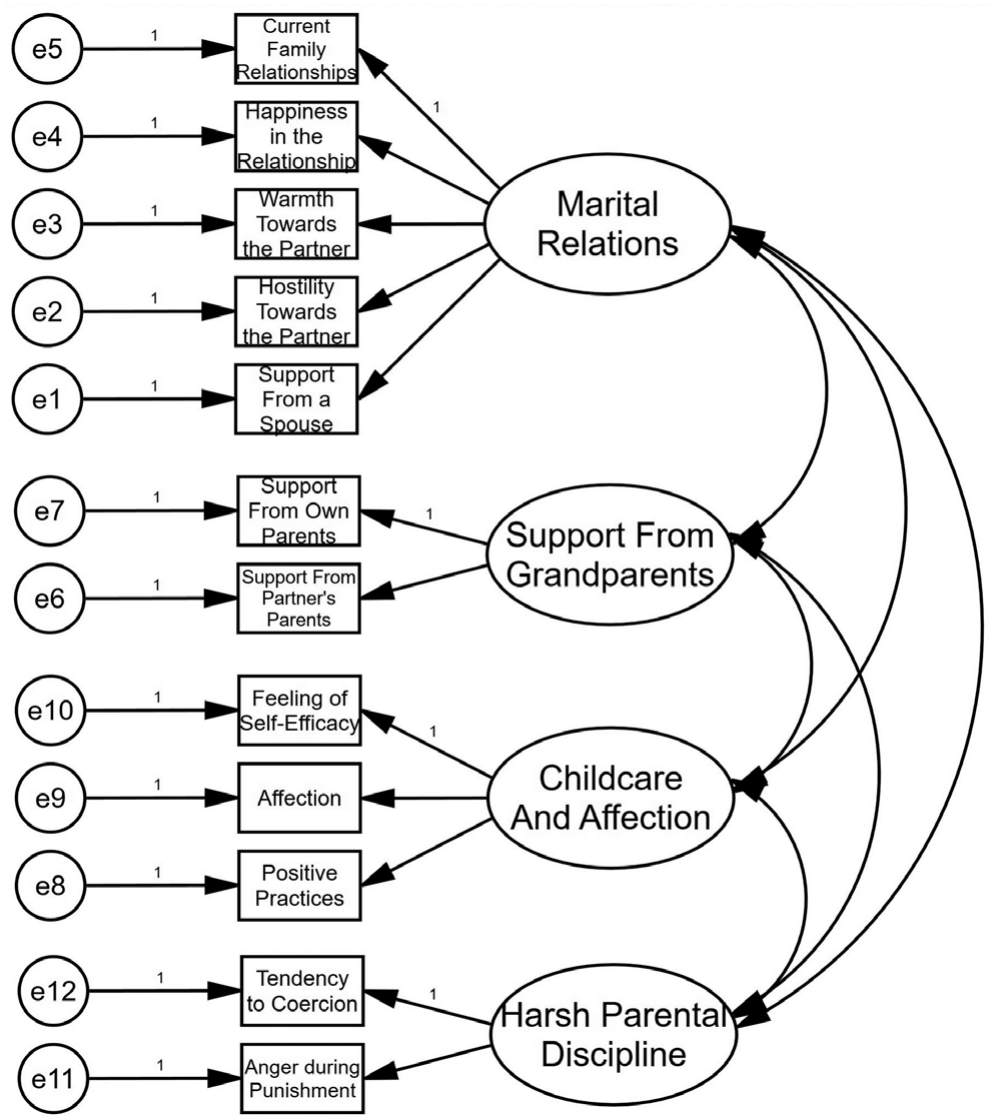
Baseline factor structure.

Therefore, for further invariance analysis we used the factor structure that was the same for both groups (Figure 2).

The results of measurement invariance testing are presented in Table 4. At the first step, the unconstrained model was tested. The results indicated acceptable model fit, suggesting good configural invariance. Then the metric invariance was assessed, which showed the non‐invariance of the suggested model between the groups (significant *χ* ^2^ change and CFI change higher than 0.01). Therefore, the partial metric invariance was tested, by subsequent relaxing of loadings constrained to be equal for the groups. Partial metric invariance was achieved after the loadings of three scales were released: *warmth towards the partner*; *support from a spouse*; *feeling of self‐efficacy*. At the next step, we assessed scalar invariance of the model, which at the previous step demonstrated partial metric invariance. The results indicated low model fit, but the partial scalar invariance was achieved after relaxing the equality constraints of the three intercepts: *current family relationships*, *support from own parents* and *affection*. As the invariant structure of the ‘marital relations’ composite included only two scales, describing perceived happiness in marital relations and reversed hostility towards the partner, rather than marital relations in general, we decided to rename it to *‘*marital well‐being’ which better reflects its meaning.

**TABLE 4:** Measurement invariance across mothers and fathers groups.

Model	*χ* ^2^ (*df*)	CFI	RMSEA	Model comparison	Δ*χ* ^2^ (Δ*df*)	ΔCFI	ΔRMSEA	Decision	Relaxed scales
M1: Configural Invariance	259.51 (96)**	0.93	0.05	—	—	—	—	—	—
M2: Metric Invariance	304.87 (104)**	0.92	0.05	M1	45.36 (8)**	0.02	0.00	Reject	—
M2a: Partial Metric Invariance	269.27 (101)**	0.93	0.05	M1	9.76 (5)	0.00	0.00	Accept	1.3(a) Warmth towards the partner 1.4 Support from a spouse 2.1(a) Feeling of self‐efficacy
M3: Scalar Invariance	464.63 (106)**	0.85	0.07	M2a	195.36** (5)	0.08	0.02	Reject	—
M3a: Partial Scalar Invariance	274.04 (103)**	0.93	0.05	M2a	4.77 (2)	0.00	0.00	Accept	1.1 Current family relationships 1.5(a) Support from own parents 2.1(d) Affection

** *p* < 0.01.

Given that only partial scalar invariance was achieved, only two invariant composite measures were calculated, that included the following scales: F1—marital well‐being (*happiness in the relationship*; *hostility towards the partner* [reversed]); F2—harsh parental discipline (*tendency to coercion* and *anger during punishment*). Scales *support from spouse*'*s parents* and *positive practices* showed good invariance, and were the only scales that remained in the corresponding factors (‘support from grandparents’ and ‘childcare and affection’, respectively). Scales *perception of impact*, *perception of the child's qualities*, *parental overprotection* and *repeated discipline* were excluded from the EFA and CFA models, therefore, they were not assessed in measurement invariance models. Table 5 presents descriptive statistics for the measures that showed measurement invariance for mothers and fathers, and for scales that were excluded at different steps of measurement invariance testing.

**TABLE 5:** Descriptive statistics for measures of family relations and parenting.

Scales	Descriptive statistics						Means comparisons	
	Group	Mean (SD)	Median	Skewness	Kurtosis	Range	*p*	Effect size
Invariant composite measures								
F1. Marital well‐being	M	24.99 (5.09)	26.00	−0.59	−0.26	10.00–35.00	0.000	0.23
	F^a^	27.20 (4.42)	28.00	−1.21	2.35	7.00–35.00		
F2. Harsh parental discipline	M	19.25 (12.91)	18.00	0.78	0.24	1.00–66.00	0.000	0.42
	F	14.07 (11.52)	12.00	1.12	1.14	1.00–55.00		
Invariant scales								
7. Support from spouse's parents	M	12.48 (5.64)	12.00	0.73	−0.10	5.00–29.00	0.000	0.62
	F	15.96 (5.81)	16.00	0.03	−0.77	5.00–29.00		
14. Positive practices	M^a^	19.30 (1.13)	20.00	−1.72	2.66	14.00–20.00	0.000	0.45
	F	17.09 (2.90)	18.00	−1.21	1.95	4.00–20.00		
Scales excluded from the EFA model								
9. Perception of impact	M	38.96 (9.04)	40.00	−0.82	0.18	8.00–50.00	0.027	0.15
	F	37.69 (10.81)	40.00	−0.88	0.29	0.00–50.00		
13. Perception of the child's qualities	M	31.13 (6.51)	32.00	−0.81	0.33	8.00–40.00	0.001	0.11
	F^a^	32.41 (6.71)	33.00	−1.17	2.25	0.00–40.00		
16. Repeated discipline	M	2.55 (1.29)	2.00	0.36	−1.03	1.00–5.00	0.000	0.29
	F	2.19 (1.18)	2.00	0.59	−0.82	1.00–5.00		
Scales excluded from the CFA model								
12. Parental overprotection	M	27.15 (9.98)	27.00	−0.07	−0.52	3.00–50.00	0.000	0.55
	F	21.30 (10.55)	21.00	0.22	−0.59	0.00–48.00		
Scales relaxed to achieve partial metric invariance								
3. Warmth towards the partner	M	29.47 (7.14)	29.00	−0.15	−0.84	10.00–42.00	0.000	0.24
	F	31.14 (6.89)	32.00	−0.36	−0.60	11.00–42.00		
5. Support from a spouse	M	34.57 (11.77)	37.00	−0.76	−0.09	0.00–50.00	0.000	0.50
	F^a^	44.80 (6.37)	47.00	−1.79	3.85	14.00–50.00		
8. Feeling of self‐efficacy	M^a^	50.30 (6.68)	51.00	−0.82	2.30	11.00–60.00	0.000	0.35
	F	44.22 (9.09)	45.00	−0.41	−0.08	15.00–60.00		
Scales relaxed to achieve partial scalar invariance								
1. Current family relationships	M	38.85 (5.33)	39.00	−0.39	0.05	22.00–48.00	0.000	0.24
	F	37.71 (4.58)	38.00	−0.64	0.52	21.00–46.00		
6. Support from own parents	M	17.37 (5.47)	18.00	−0.20	−0.54	5.00–29.00	0.000	0.34
	F	15.71 (5.48)	15.00	0.23	−0.47	5.00–29.00		
11. Affection^a^	M	47.27 (3.98)	49.00	−1.89	3.72	28.00–50.00	0.000	0.52
	F	44.26 (6.70)	47.00	−1.48	2.09	15.00–50.00		

*Note:* Effect size = Cohen's *d*.

^a^ The non‐parametric test was used. Other comparisons were performed with *t*‐test.

### 3.2 Associations Among Marital Relations and Parenting for Mothers and Fathers

Table 6 presents correlations among all measures, separately for mothers and fathers.

**TABLE 6:** Between‐measure correlations for mothers' and fathers' scores.

	Father →													
Mother ↓	1	3	F1	5	6	7	8	9	11	12	13	14	16	F2
1. Current family relationships	0.56***	0.59***	0.55***	0.37***	0.13*	0.12*	0.26***	0.29***	0.28***	0.06	0.15**	0.20***	−0.07	−0.26***
3. Warmth towards the partner	0.60***	0.44***	0.61***	0.53***	0.19**	0.22***	0.48***	0.16**	0.47***	0.22***	0.17**	0.34***	−0.02	−0.30***
F1. Marital well‐being	0.67***	0.66***	0.47***	0.52***	0.12*	0.16**	0.28***	0.15**	0.36***	0.05	0.07	0.23***	−0.14*	−0.35***
5. Support from a spouse	0.55***	0.45***	0.48**	0.30***	0.16**	0.10	0.28***	0.13*	0.31***	0.07	0.13*	0.25***	−0.07	−0.25***
6. Support from own parents	0.16**	0.13*	0.10	0.06	0.34***	0.46***	0.21***	−0.03	0.08	0.04	0.04	0.18**	0.01	−0.16**
7. Support from spouse's parents	0.22***	0.18**	0.15**	0.25***	0.31***	0.15*	0.17**	0.01	0.16**	0.05	−0.02	0.12*	0.00	−0.11
8. Feeling of self‐efficacy	0.17**	0.35***	0.20***	0.09	0.08	0.02	0.25***	0.15**	0.61***	0.23***	0.29***	0.40***	−0.04	−0.34***
9. Perception of impact	0.08	−0.01	0.04	0.01	0.00	−0.04	−0.01	0.30***	0.21***	0.02	0.10	0.17**	0.04	−0.16**
11. Affection	0.07	0.24***	0.09	0.09	0.23***	0.08	0.49***	0.12*	0.30***	0.38***	0.39***	0.49***	−0.18**	−0.43***
12. Parental overprotection	0.07	0.19**	0.08	0.00	0.18**	0.07	0.18**	−0.08	0.29***	0.22***	0.25***	0.19***	−0.04	−0.11
13. Perception of the child's qualities	−0.01	0.08	0.03	−0.02	0.15**	0.07	0.32***	0.06	0.31***	0.24***	0.34***	0.21***	−0.07	−0.24***
14. Positive practices	0.17**	0.23***	0.17**	0.17**	0.10	0.03	0.26***	0.11*	0.39***	0.18**	0.24***	0.11*	0.12*	−0.30***
16. Repeated discipline	−0.04	−0.02	−0.06	−0.03	−0.01	0.03	−0.02	−0.07	0.06	−0.01	−0.01	0.04	0.28***	0.20***
F2. Harsh parental discipline	−0.14*	−0.32***	−0.31***	−0.02	−0.12*	0.00	−0.30***	−0.02	−0.25***	−0.20***	−0.13*	−0.18**	0.12*	0.25***

*Note:* *Correlation is significant at the 0.05 level (2‐tailed); **correlation is significant at the 0.01 level (2‐tailed), ***correlation is significant at the 0.001 level (2‐tailed). Correlations are presented without correction for multiple testing. Mothers' measures are presented below the diagonal. Fathers' measures are presented above the diagonal. Within‐couple correlations (mother–father) for the same measures are presented on the diagonal and indicated in grey.

For both parents, all measures of marital relations were significantly positively inter‐correlated, and weak significant correlations were observed for grandparents' support and parenting, with some inconsistencies between parents.

For both parents, current family relations weakly positively correlated with self‐efficacy and positive parenting, negatively with harsh parenting. For fathers, current family relations were also significantly associated with all measures of parenting, except for overprotection and repeated discipline.

For fathers, warmth towards the partner was significantly associated with all measures of parenting, except for repeated discipline. For mothers, the correlations of warmth with perceived impact and perception of child qualities were non‐significant.

Mothers' marital well‐being showed weak significant correlations with their self‐efficacy and positive parenting, negative with harsh parental discipline. For fathers, marital well‐being was significantly associated with all measures of parenting, except for overprotection and perception of child's qualities.

The only measure of parenting that showed positive significant correlations with spousal support for mothers was positive parenting. However, for fathers, perceived support from a spouse was significantly positively associated with self‐efficacy, perceived impact, affection and positive parenting; and negatively—with repeated discipline and harsh parenting.

Support mothers received from their parents was weakly positively associated with their perception of current family relationships, warmth towards the partner, affection, overprotection, perception of child's qualities and negatively—with harsh parenting. Support they received from partner's parents significantly positively correlated with all their measures of marital relations. For fathers, perceived support from their parents significantly positively correlated with all measures of marital relations, as well as with affection, overprotection and perception of child's qualities; and negatively—with harsh parenting. Support fathers received from partner's parents was associated with current family relationships, warmth towards the partner and marital well‐being. The associations with parenting were non‐significant.

### 3.3 Mother–Father Agreement and Cross‐Measure Associations

The cross‐parent‐cross‐measure correlational matrix is presented in Table A2. All corresponding measures correlated significantly between the parents, although the correlations for measures of parenting were weak. Cross‐measure, for both parents, marital well‐being was significantly, but weakly, positively related to partner's self‐efficacy in parenting and positive parenting; and negatively—to harsh parenting. In addition, mothers' perception of current marital relations and marital well‐being weakly positively correlated with fathers' perceived impact and affection. Maternal warmth towards the spouse was significantly associated with all paternal measures of parenting, except for perceived impact and repeated discipline. Support mothers received from the spouse weakly significantly correlated with all paternal measures of parenting, except for perception of infant's qualities. The weak correlations with paternal perception of mothers' support were significant only for mothers' self‐efficacy and harsh discipline. For both parents, support they received from child's grandparents did not correlate significantly with their partners' parenting. However, for mothers, support from partner's parents weakly correlated with fathers' perception of marital relationships; for fathers—with maternal warmth towards the spouse and marital well‐being.

### 3.4 Average Differences Between Mothers and Fathers

All comparisons between mothers and fathers were significant with weak to moderate effect (Table 5). Fathers on average expressed higher levels of marital well‐being, more warmth towards the spouse and perceived higher levels of support. Mothers were more satisfied with an emotional aspect of these relationships, perceiving them as overall ‘healthier’, characterised by higher levels of acceptance, support and collaboration. For both parents, support from maternal parents was rated higher than from paternal parents.

For further ANCOVA analysis, the measure of differences in the perception of current family relationships was calculated as mothers' minus fathers' scores on *current family relationships*. The resulting measure varied from −17 (fathers reporting more positive relations) to 16 (mothers reporting more positive relations), with the average differences *M* = 1.19, SD = 4.70—indicating that mothers on average perceived family relations as more positive. Average differences calculated on absolute values (i.e., the modulus of the differences) were *M* = 3.74, SD = 3.09. In the model, the raw (both positive and negative) data were included. The results of ANCOVA are presented in Table A3. The Levene's test was significant for *marital well*‐*being* (*p* = 0.001) and *current family relationships* (*p* = 0.012) models. However, given that the sample sizes of mothers and fathers was similar, and the variance ratio was 1.3 in both cases, we proceeded with means comparison (Blanca et al. 2018). The covariate was significant for all three comparisons, indicating that differences in parents' perceptions of family relationships are partially explained by the disagreement in the perception of family situation. The differences between parents remained significant when controlled for the covariate, with similar effect size.

Scores of most parenting measures were higher for mothers than for fathers, including harsh parenting. Specifically, compared with fathers, mothers reported higher overprotection and general affection to the child, and perceived themselves as more effective as a parent. However, in general they expressed lower self‐control when the child was particularly fussy. Compared with mothers, fathers perceived the qualities of their children as more special, unique and on average better than those of other children.

## 4 Discussion

The results of the study showed that the measures of family relations and parenting were organised into coherent factor‐based groupings, suggesting that they represent several underlying latent constructs. Measures of marital relations and support formed two factors in both mothers and fathers, as expected from the content of the scales. The first factor reflected perception of marital relations quality. The second factor included measures of financial, instrumental and emotional support provided by infant's maternal and paternal grandparents. The third and the fourth factors described childcare and use of harsh parental discipline, respectively.

Although this structure was similar for mothers and fathers, some differences emerged in the structure of family relations and parenting between mothers and fathers. The partially invariant structure included *happiness in the relationship* and reversed *hostility towards the partner* as the measures of marital well‐being; *support from spouse*'*s parents* as a measure of grandparents' support; *positive practices* as a measure of childcare and affection towards the infant; *tendency to coercion* and *anger during punishment* as the measures of harsh parental discipline. The results add to the limited research on the invariance of measures assessing marital relations and parental behaviours. Specifically, previous research has also observed partial invariance of parenting measures, including parental acceptance, psychological intrusiveness, harshness (Adamsons and Buehler 2007) and parental warmth (Crapo et al. 2021). Previous studies also found invariance of marital relations quality (South, Krueger, and Iacono 2009), parental autonomy support (Hughes, Lindberg, and Devine 2018) and parental beliefs on child's independence (Crapo et al. 2021). Therefore, a complex picture of similarities and differences between mothers and fathers is emerging in the literature, and calls for further research to understand the causes of the inconsistencies across the studies.

Analyses of within‐parent associations across measures of family relations showed weak but significant associations between marital relations, grandparents' support and parenting. For mothers, spousal support was weakly associated with only positive parenting, whereas for fathers it was associated with most characteristics of parenting. It is possible that this can be explained by the differences in the amount of support provided by the spouses: for mothers perceived support was significantly lower than for fathers.

The analyses of inter‐parental agreement revealed moderate between‐parent (within‐couple) correlations for all measures of marital relations, consistent with previous research indicating that mothers and fathers perceive most aspects of family relations similarly (McCoy et al. 2013; Queen et al. 2013; Pisula and Porębowicz‐Dörsmann 2017). The measure of family relations that showed the lowest within‐couple correlation was spouse's parents' support. This was expected, as husbands and wives were rating support from different relatives: husband—of his wife's parents and wife—of her husband's parents, with potential differences in their geographical and other limitations for supporting their family.

In contrast to measures of marital relations, measures of parenting were largely parent‐specific: all inter‐couple correlations were significant but weak. The correlations for most parenting measures were similar to those reported by Boivin et al. (2000). However, in our study, the within‐couple correlation for overprotection was significantly weaker, probably indicating some cultural differences. The lowest correlation was observed for positive parenting, demonstrating that maternal and paternal involvement were largely different (Laflamme, Pomerleau, and Malcuit 2002; Siqveland et al. 2022). These results suggest that parenting behaviours and attitudes of one parent are largely independent of their spouse's parenting, and are likely to reflect individual differences in personality, values and parents' childhood experiences (Fulu et al. 2017; Franklin‐Luther and Volk 2021). Previous research has also suggested that some differences in parental involvement in childcare result from cultural gender role expectations, as well as their postpartum employment status (Giallo et al. 2013; Shwalb and Shwalb 2014)—factors that we did not explore in the present study.

Family relations were weakly associated with parenting, both within‐parent and between‐parents. This adds to the previous research that found within‐parent associations among perceived family relations and parenting (Krishnakumar and Buehler 2000; Lee and Doherty 2007; Bernier, Jarry‐Boileau, and Lacharité 2014), as well as between‐parent associations between paternal behaviour in marital conflict and maternal parenting behaviours (Gao et al. 2019).

Finally, the study examined average differences in perceptions of family processes between mothers and fathers. The results regarding the marital relations were somewhat contradictory. Fathers were in general happier in the relations, more satisfied with partners' support and expressed more warmth and care, than mothers. At the same time, they perceived the interaction within the family as less accepting, safe and empathetic. This may be related to greater difficulties of fathers than mothers with emotional expressions, viewed as contradictory to ‘masculine’ behaviour in some cultures, including in Russia, where the study was conducted (Kerig, Alyoshina, and Volovich 1993; Levant 2011). Lower levels of family relationship satisfaction observed in mothers may be due to high emotional and physical pressures caused by motherhood (Dew and Wilcox 2011; Bower et al. 2013). The results also showed that support provided by maternal grandparents was higher than that by paternal grandparents, which is in line with previous research (Statham 2011).

We also explored whether the observed average differences were moderated by the degree of parental agreement in their evaluations of current family relationships. Although the effect of the covariate was significant, adding it led to no significant changes in the effect size of differences between the parents in marital well‐being, warmth and current family relationships. This implies that although discrepancy between the spouses' evaluations plays a role, it does not account for the observed average differences. Other individual, relational or contextual factors underlying these differences need to be explored. Previous studies suggested that discrepancies in parents' perceptions may at least partly explain why full mother–father measurement invariance is not observed for some measures of different aspects of marital relations.

Average mother–father differences were also found for most characteristics of parenting, which were higher for mothers. This is consistent with previous research and may reflect a stronger bond between mothers and the child during this period (e.g., Foley and Hughes 2018). At the same time, unlike the results from previous research (Boivin et al. 2000, 2005), in our study, mothers demonstrated a higher tendency to coercion, than fathers, suggesting that mothers on average experienced higher difficulties in self‐control when the child demonstrated unwanted behaviour. Some previous studies found a decrease in self‐control in mothers after the childbirth (van Scheppingen, Denissen, and Bleidorn 2018), which may be related with greater emotional problems during this time (Boivin et al. 2000), which may be related to greater emotional problems during this time (Boivin et al. 2000, 2005).

Our results also showed that fathers tended to admire the child more, compared with mothers, which is reflected in greater perception of infant's qualities. It is possible that these differences between parents may be explained by differences in parental involvement, time spent with the child in different contexts and differences in parental expectations (Lewis and Lamb 2003). Moreover, these differences may be moderated by culture. Indeed, previous research form a Canadian sample did not find significant differences in this dimension between mothers and fathers (Boivin et al. 2000).

The study had a number of limitations. First, the data were self‐reported, and therefore, the answers may have been influenced by social desirability and other biases. Second, the attrition rate from the previous research wave, conducted during the pregnancy, was 35%, which may undermine the study's generalisability. For example, more participants experiencing problems in the relationships might have withdrawn from the study. Third, although the PLIS sample includes two groups of families (natural conception and ART), the potential differences related to conception method were not explored in the present analyses. The ART group was smaller than naturally conceiving group, resulting in lack of power for establishing weak associations and for comparing associations across the groups. Also, only married couples participated in the study, which limits the generalisability of the results.

Despite these limitations, the study provided new insights into the family relations processes. The findings inform further research that needs to take into account the non‐invariance of some aspects of marital relations and parenting between mothers and fathers, likely resulting from the differences in their perceptions and experiences. The study also found that mother–father correlations were moderate for family relations perceptions but negligible for parenting. The established patterns of associations among different constructs of family relations and parenting may be useful for family counselling. Future research is needed to assess how family processes develop over time and how they are linked to child development.

## Conflicts of Interest

The authors declare no conflicts of interest.

## Data Availability

The data are available from the corresponding author upon reasonable request.

## Appendix A

**TABLE A1:** Descriptive statistics for measures of family relations and parenting (mothers and fathers).

Scales	Group	Mean (SD)	Median	Skewness	Kurtosis	Range
1. Current family relationships	M	38.85 (5.33)	39.00	−0.39	0.05	22.00–48.00
	F	37.71 (4.58)	38.00	−0.64	0.52	21.00–46.00
2. Happiness in the relationship	M	5.16 (1.30)	5.00	−0.62	−0.12	1.00–7.00
	F	5.66 (1.22)	6.00	−0.87	0.48	1.00–7.00
3. Warmth towards the partner	M	29.47 (7.14)	29.00	−0.15	−0.84	10.00–42.00
	F	31.14 (6.89)	32.00	−0.36	−0.60	11.00–42.00
4. Hostility towards the partner	M	12.21 (4.24)	12.00	0.56	−0.29	4.00–25.00
	F	10.46 (3.83)	10.00	1.11	1.70	4.00–27.00
5. Support from a spouse	M	34.57 (11.77)	37.00	−0.76	−0.09	0.00–50.00
	F^a^	44.80 (6.37)	47.00	−1.79	3.85	14.00–50.00
6. Support from own parents	M	17.37 (5.47)	18.00	−0.20	−0.54	5.00–29.00
	F	15.71 (5.48)	15.00	0.23	−0.47	5.00–29.00
7. Support from spouse's parents	M	12.48 (5.64)	12.00	0.73	−0.10	5.00–29.00
	F	15.96 (5.81)	16.00	0.03	−0.77	5.00–29.00
8. Feeling of self‐efficacy	M^a^	50.30 (6.68)	51.00	−0.82	2.30	11.00–60.00
	F	44.22 (9.09)	45.00	−0.41	−0.08	15.00–60.00
9. Perception of impact	M	38.96 (9.04)	40.00	−0.82	0.18	8.00–50.00
	F	37.69 (10.81)	40.00	−0.88	0.29	0.00–50.00
10. Tendency to coercion	M	17.73 (12.38)	16.00	0.76	0.26	0.00–63.00
	F	12.67 (11.11)	10.00	1.12	1.16	0.00–52.00
11. Affection^a^	M	47.27 (3.98)	49.00	−1.89	3.72	28.00–50.00
	F	44.26 (6.70)	47.00	−1.48	2.09	15.00–50.00
12. Parental overprotection	M	27.15 (9.98)	27.00	−0.07	−0.52	3.00–50.00
	F	21.30 (10.55)	21.00	0.22	−0.59	0.00–48.00
13. Perception of the child's qualities	M	31.13 (6.51)	32.00	−0.81	0.33	8.00–40.00
	F^a^	32.41 (6.71)	33.00	−1.17	2.25	0.00–40.00
14. Positive practices	M^a^	19.30 (1.13)	20.00	−1.72	2.66	14.00–20.00
	F	17.09 (2.90)	18.00	−1.21	1.95	4.00–20.00
15. Anger during punishment	M	1.63 (0.82)	1.00	1.21	0.79	1.00–4.00
	F^a^	1.36 (0.66)	1.00	1.98	3.81	1.00–4.00
16. Repeated discipline	M	2.55 (1.29)	2.00	0.36	−1.03	1.00–5.00
	F	2.19 (1.18)	2.00	0.59	−0.82	1.00–5.00

Abbreviations: F = father; M = mother.

^a^ The scales with non‐normal distribution.

**TABLE A2:** Correlations between mothers' and fathers' scores for each measure.

	Father →													
Mother ↓	1	3	F1	5	6	7	8	9	11	12	13	14	16	F2
1. Current family relationships	0.56***	0.41***	0.43***	0.32***	0.10	0.09	0.20***	0.24***	0.15**	0.03	0.07	0.19**	0.02	−0.20***
3. Warmth towards the partner	0.39***	0.44***	0.41***	0.31***	0.08	0.12*	0.20***	0.10	0.19**	0.19**	0.12*	0.15**	0.01	−0.18**
F1. Marital well‐being	0.47***	0.35***	0.47***	0.28***	0.11	0.13*	0.12*	0.16**	0.14*	−0.03	0.03	0.13*	0.01	−0.17**
5. Support from a spouse	0.38***	0.40***	0.34***	0.30***	0.12*	0.10	0.25***	0.26***	0.23***	0.13*	−0.01	0.39***	0.13*	−0.12*
6. Support from own parents	0.07	0.08	0.08	0.01	0.34***	0.63***	0.10	−0.02	0.11	0.07	0.06	0.02	0.00	−0.06
7. Support from spouse's parents	0.11*	0.05	0.07	0.09	0.54***	0.15*	0.07	−0.01	0.00	0.06	0.03	0.10	0.04	0.02
8. Feeling of self‐efficacy	0.05	0.24***	0.16**	0.20***	0.06	0.05	0.25***	0.02	0.23**	0.18**	0.25***	0.10	−0.09	−0.27***
9. Perception of impact	0.01	−0.03	0.01	−0.08	0.02	0.04	−0.04	0.30***	0.04	−0.07	0.12*	0.00	−0.05	−0.09
11. Affection	0.06	0.12*	0.07	0.08	0.09	0.10	0.14**	0.04	0.30***	0.21***	0.27***	0.10	−0.07	−0.20***
12. Parental overprotection	0.03	0.08	0.08	0.10	0.09	0.11	0.06	−0.09	0.13*	0.22***	0.18**	0.07	−0.04	−0.13*
13. Perception of the child's qualities	0.00	0.03	−0.02	−0.01	0.08	0.03	0.02	−0.06	0.10	0.08	0.34***	0.14*	0.05	−0.04
14. Positive practices	0.11*	0.16**	0.22**	0.11	0.04	0.07	0.08	0.04	0.17**	0.11	0.07	0.11**	−0.03	−0.20***
16. Repeated discipline	−0.07	−0.05	−0.06	−0.03	−0.05	−0.04	−0.01	−0.04	−0.03	0.12*	−0.05	−0.04	0.28***	0.12*
F2. Harsh parental discipline	−0.09	−0.17**	−0.12*	−0.14*	−0.08	−0.13*	−0.10	0.00	−0.15**	−0.05	−0.22***	−0.04	0.07	0.25***

*Note:* *Correlation is significant at the 0.05 level (2‐tailed); **correlation is significant at the 0.01 level (2‐tailed); ***correlation is significant at the 0.001 level (2‐tailed). Correlations are presented without correction for multiple testing. Within‐couple correlations (mother–father) for the same measures are presented on the diagonal and indicated in grey.

**TABLE A3:** ANCOVA results for marital relations measures (factor—family member; covariate—differences in the perception of current family relationships).

	*df*	Mean square	*F*	*p*	Partial *η* ^2^
F1 marital well‐being					
(a) Uncorrected model					
Family member	1	822.33	36.12	0.000	0.05
Error	670	22.76			
(b) Model with the covariate included					
Family member	1	744.16	33.55	0.000	0.05
Differences in the perception of current family relationships	1	261.78	11.80	0.001	0.02
Error	625	22.18			
1. Current family relationships					
(a) Uncorrected model					
Family member	1	219.96	8.91	0.003	0.01
Error	680	22.77			
(b) Model with the covariate included					
Family member	1	226.11	9.32	0.002	0.01
Differences in the perception of current family relationships	1	352.00	14.50	0.000	0.02
Error	639				
3. Warmth towards the partner					
(a) Uncorrected model					
Family member	1	481.10	9.76	0.002	0.01
Error	680	49.27			
(b) Model with the covariate included					
Family member	1	410.97	8.49	0.004	0.01
Differences in the perception of current family relationships	1	280.08	5.79	0.016	0.01
Error	634				

*Note:* Differences in the perception of current family relationships were calculated as mothers' minus fathers' score.
